# Relay Selection for Dual-Hop Cooperative Ambient Backscatter Communication Systems

**DOI:** 10.3390/s23135791

**Published:** 2023-06-21

**Authors:** Ping Yang, Weichao Kuang, Shanjin Wang

**Affiliations:** School of Electrical Engineering and Intelligentization, Dongguan University of Technology, Dongguan 523808, China

**Keywords:** Internet of Things, ambient backscatter communication (AmBC), cooperative receiver, relay selection, decoded and forward (DF), outage probability (OP)

## Abstract

Previous works only focus on the optimization design for the dual-hop cooperative ambient backscatter communication (AmBC) system with single-relay selection. The impact of relay selection on the outage performance of dual-hop cooperative AmBC systems is still missing. Motivated by this, in this paper, we investigate the outage performance of a dual-hop cooperative AmBC system with single-relay selection, where the backscatter link shares the receiver with the cellular link and the harmful direct-link interference (DLI) is mitigated by using successive interference cancellation (SIC). In the system considered, the selected relay has dual functions. One is to forward message for the cellular link, and the other is to act as the radio-frequency (RF) source for the backscatter device (BD). Specifically, after proposing two novel single-relay selection schemes (RSSs), namely reactive RSS and proactive RSS, we derive the closed-form outage probability (OP) expressions for both RSSs, which can be performed in a distributed manner. To gain more insights, the asymptotic OPs at high signal-to-noise ratio (SNR) are explored and the outage performance comparison between the reactive RSS and proactive RSS are also provided. Results show that the proposed reactive RSS is outage-optimal among all possible single-relay selection schemes. The theoretical analysis is validated by Monte Carlo simulations. The results also show that the relay selection scheme, the number of relays, the location of BD, and the reflection coefficient of BD have great impact on the outage performance of cooperative AmBC systems.

## 1. Introduction

Ambient backscatter communication (AmBC) is regarded as an efficient technology to solve the spectrum scarcity and limited battery capacity problem for next-generation Internet of Things (IoT) [1,2]. In AmBC systems, the backscatter link shares the same spectrum and radio-frequency (RF) source with the primary link, i.e., cellular or WiFi systems. In particular, the backscatter device (BD) modulates its message on the incident primary signals and reflects the modulated signals to its associated receiver without requiring high-cost and power-hungry active components (e.g., oscillators, up-converters, power amplifiers, and filters) [3].

Owing to the spectrum sharing nature between the primary system and the backscatter system, traditional AmBC may suffer from severe direct-link interference (DLI) from the primary transmitter, resulting in unacceptable performance degradation for backscatter transmission. One way to tackle the DLI problem is to shift the backscatter signals to a frequency band different from the one used by the primary system [4,5,6,7]. However, additional spectrum is required for the above approach, which may not be suitable for massive IoT connections. Cooperative AmBC, also referred to as symbiotic radio (SR), which integrates the backscatter receiver with the primary receiver, is another spectrum efficient way to tackle the DLI problem [8,9,10,11,12]. In a cooperative AmBC system, the harmful DLI is mitigated by using successive interference cancellation (SIC), i.e, the receiver decodes the primary signal first and then subtract it from the received signal when decoding the BD signal. The optimal maximum-likelihood (ML) detector design for a cooperative AmBC system was investigated in [8]. The results in [8] showed that the SIC-based detectors can achieve near-ML detection performance. The outage probability and ergodic rate for a cooperative AmBC system were studied in [9,10], respectively. The transmit beamforming optimization problems for multiple-input single-output (MISO) and multiple-input multiple-output (MIMO) cooperative AmBC systems were investigated in [11,12], respectively.

Recently, the concept of cooperative AmBC was introduced to full-duplex communication systems [13], non-orthogonal multiple access (NOMA) systems [14,15,16,17], reconfigurable intelligent-surface (RIS) -aided systems [18,19], and physical layer security systems [20], respectively. In [16], the exact and asymptotic outage probabilities of a downlink NOMA multiplexing based cooperative AmBC system over Nakagami-m fading channels were derived. In [17], the authors proposed an energy efficient optimization framework that maximizes the energy efficiency of a AmBC-assisted cooperative NOMA IoT system. Inspired by RIS’s low-cost and reconfigurability nature, the RIS-assisted AmBC network has been a concern of many scholars. For a single-user RIS-aided AmBC network, the authors in [18] studied a transmit power minimization problem. For multiuser RIS-aided MISO AmBC networks, the authors in [19] investigated a resource allocation problem to maximize the system energy-efficiency by jointly optimizing the active beamforming of the primary transmitter and the passive beamforming of the RIS. Due to the broadcast property, the backscattered signals by BDs may be wiretapped by eavesdroppers. Physical layer security is regarded as a promising solution to improve network security. In [20], the closed-form expressions of secrecy outage probability (SOP) are derived for a cooperative AmBC-based intelligent transportation system with a friendly jammer.

However, the authors of [8,9,10,11,12,13,14,15,16,17,18,19,20] focus on one-hop cooperative AmBC systems. In fact, when backscatter devices are located far away from the primary transmitter, due to the severe wireless channel fading, conventional one-hop transmissions may not work well. On the one hand, as mentioned in many references, using relays can provide extra spatial diversity and expand the coverage of wireless communication systems [21,22]. Recently, the relay-based one-way and two-way cooperative AmBC systems were investigated in [23,24,25,26,27,28,29,30], respectively. In particular, the authors in [23] studied the outage probability and throughput of an energy harvesting enabled decode-and-forward (DF) cognitive relay network over Rayleigh fading channels, where AmBC was utilized as secondary communication. Later, this work was extended to Nakagami-m fading channels and nonlinear energy harvesting model [24,25], respectively. In [26], an optimal time allocation scheme was proposed to maximize the throughput for a DF-enabled cooperative AmBC system. The authors in [27] proposed an opportunistic ambient backscatter assisted DF relaying scheme, where the relay can forward signals and perform AmBC operation concurrently. In [28], a dual-hop cooperative AmBC system was considered, where the AmBC technique was used in the first hop. In [29], the secrecy performance and energy efficiency of a relay-based cooperative AmBC system with passive eavesdroppers were investigated. In [30], the authors evaluated the outage performance of an opportunistic source selection based two-way cooperative AmBC system.

The aforementioned works assume that only a single relay was involved. However, multi-relay networks are more general in practical situations and single-relay selection is a simple technique to improve the performance of cooperative relay systems [31,32]. To the best of our knowledge, the relay selection for multi-relay cooperative AmBC systems has been recently considered only in [33,34]. In [33], the scheme of jointly optimizing power allocation and relay selection for a DF-enabled cooperative AmBC system was investigated. In [34], the authors studied the throughput maximization problem by jointly optimizing the time allocation and relay selection.

However, the authors of [33,34] focus their attention on the optimization design and the optimal relay is selected only by the iterative algorithm. The closed-form expression for the optimal relay selection criteria is not provided and the outage performance of the multi-relay cooperative AmBC system is not investigated in [33,34]. In this work, on the other hand, we investigate the impact of relay selection on the outage performance of dual-hop cooperative AmBC systems. Although a number of single-relay selection schemes (RSSs) in conventional cooperative networks have been proposed in the literature [31,32,35,36], e.g., for conventional reactive and proactive single RSSs, existing work has focused on the scenario where only a type of link is involved and the same targeted data rate at each hop is assumed. Nevertheless, there are two different types of links and different targeted data rates for the cellular link and backscatter link are utilized in the considered system. Thus, the conventional single-relay selection schemes cannot be adopted in the considered system directly. Taking into account such differences, two novel single RSSs, namely reactive RSS and proactive RSS, are proposed in this paper. The aim of reactive RSS is to maximize the rate of the backscatter link provided the cellular user’s link quality can be guaranteed. For the proactive RSS, we regard the backscatter link as a virtual hop (the third hop) in the cellular system to circumvent the two different types of link problems and resort to a scale method to tackle the problem that different targeted data rates are involved. More precisely, the main contributions and novelties of this paper are summarized as follows:

- Different from the conventional single-relay selection schemes in [31,32], where only a type of link is involved, there are two different types of links in our considered system, i.e., the cellular link and backscatter link. Thus, the conventional single-relay selection schemes cannot be adopted in the considered system straightforwardly. In this paper, considering the two different types of links, we proposed two novel single-relay selection schemes (RSSs), namely reactive RSS and proactive RSS, which are appropriate for dual-hop cooperative AmBC systems and can be performed in a distributed manner. Moreover, theoretical results show that the proposed reactive relay selection scheme is outage-optimal among all possible single-relay selection schemes.
- The closed-form expressions of the outage probability (OP) for the proposed relay selection schemes are derived and the asymptotic OPs are obtained at high signal-to-noise ratio (SNR). The outage performance comparison between the reactive RSS and proactive RSS is also provided to gain more insight.
- Both theoretical and simulation results demonstrate that an outage floor phenomenon occurs at high SNR due to the interference caused by the backscatter link and the outage floor can be reduced by increasing the number of relays or decreasing the reflection coefficient of the backscatter device.

The paper is organized as follows. The system model is given in Section 2. Section 3 focuses on the proposed relay selection schemes in multi-relay cooperative AmBC systems. Theoretical OP derivations and OP comparison between different schemes are provided in Section 4. Section 5 deals with the asymptotic analysis. Numerical results are presented in Section 6. Section 7 concludes the paper.

## 2. System Model

**Figure 1:**
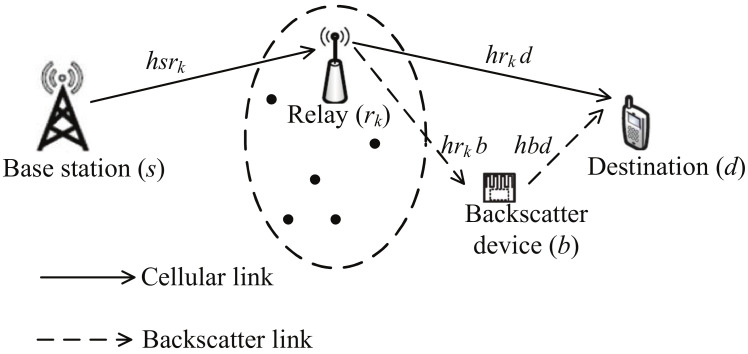
System model.

Consider a cooperative AmBC system with the coexistence of cellular and backscatter links [33,34], as illustrated in Figure 1. The cellular link employs opportunistic DF relaying transmission, while the backscatter link employs ambient backscatter transmission. The backscatter device (*b*) shares the same spectrum and receiver with the cellular link. The cellular link consists of a base station (*s*), *K* relays ($r_{k}\in R,R=\left\{r_{1},r_{2},\cdots ,r_{K}\right\}$), and a destination (*d*). Each node is equipped with a single antenna. We assume that there is no direct link between *s* and *d* due to obstacles. The wireless channel between node *i* (*s*, $r_{1}$, ⋯, $r_{K}$, *b*) and node *j* ($r_{1}$, ⋯, $r_{K}$, *b*, *d*) is modeled as an independent complex Gaussian random variable with zero mean and variance $1/\lambda_{ij}$, i.e., $h_{ij}∼\mathrm{CN}\left(0,1/\lambda_{ij}\right)$. Thus, the channel gain $g_{ij}={\left|h_{ij}\right|}^{2}$ follows exponential distribution with probability density function (PDF) $f_{g_{ij}}\left(x\right)=\lambda_{ij}\exp\left(-\lambda_{ij}x\right),x\geq 0$. For analysis simplicity, we assume that all relays are located close to one another (optimal clustering) [37,38], which implies $\lambda_{sr_{k}}=\lambda_{sr}$, $\lambda_{r_{k}d}=\lambda_{rd}$, and $\lambda_{r_{k}b}=\lambda_{rb}$, $r_{k}\in R$.

The whole transmission is divided into two phases. During the first phase, the base station broadcasts its signal to the *K* relays. The received signal at $r_{k}$ is given by

(1) $$y_{r_{k}}=\sqrt{P_{s}}h_{sr_{k}}x_{s}+n_{r}$$

where $x_{s}$ is the signal of *s* with unit energy ($E[|x_{s}{|}^{2}]=1$), $P_{s}$ is the transmit power of *s*, $n_{r}$ is the additive white Gaussian noise (AWGN) with zero mean and variance $\sigma_{n}^{2}$, i.e., $n_{r}∼\mathrm{CN}\left(0,\sigma_{n}^{2}\right)$. Then, the SNR at $r_{k}$ is

(2) $$\gamma_{sr_{k}}=\frac{P_{s}g_{sr_{k}}}{\sigma_{n}^{2}}=\gamma_{1}g_{sr_{k}}$$

where $\gamma_{1}=P_{s}/\sigma_{n}^{2}$ is the average SNR of the first hop.

During the second phase, one of the relays $r_{k}$ ($r_{k}\in R$) is selected to forward $x_{s}$ to *d*, and at the same time the backscatter device modulates its own signal $v_{b}$ ($E[|v_{b}{|}^{2}]=1$) over the incident signal $x_{s}$ to *d*. The received signal at *d* is

(3) $$y_{d}=\sqrt{P_{r}}h_{r_{k}d}x_{s}+\alpha \sqrt{P_{r}}h_{r_{k}b}h_{bd}x_{s}v_{b}+n_{d}$$

where $P_{r}$ is the transmit power of $r_{k}$, α∈[0,1] is the reflection coefficient of the backscatter device, and $n_{d}∼\mathrm{CN}\left(0,\sigma_{n}^{2}\right)$ is the AWGN at *d*. By using successive interference cancellation (SIC), the destination decodes $x_{s}$ first and then subtracts it from $y_{d}$ for decoding $v_{b}$. Therefore, the signal-to-interference-plus-noise ratio (SINR) to decode $x_{s}$ is

(4) $$\gamma_{r_{k}d}=\frac{P_{r}g_{r_{k}d}}{\alpha^{2}P_{r}g_{r_{k}b}g_{bd}+\sigma_{n}^{2}}=\frac{\gamma_{2}g_{r_{k}d}}{\alpha^{2}\gamma_{2}g_{r_{k}b}g_{bd}+1}$$

where $\gamma_{2}=P_{r}/\sigma_{n}^{2}$ is the average SNR of the second hop. If $x_{s}$ is decoded successfully, after SIC, the SNR to decode $v_{b}$ is

(5) $$\gamma_{r_{k}b}=\frac{\alpha^{2}P_{r}g_{r_{k}b}g_{bd}}{\sigma_{n}^{2}}=\alpha^{2}\gamma_{2}g_{r_{k}b}g_{bd}$$

From (4) and (5), it is noted that $\gamma_{r_{k}d}$, $\gamma_{r_{m}d}$, and $\gamma_{r_{k}b}$ ($k\neq m,r_{k},r_{m}\in R$) are dependent due to the common backscatter link.

## 3. Relay Selection Schemes

In this section, we will describe how reactive RSS and proactive RSS occur.

### 3.1. Reactive RSS

The aim of reactive RSS is to maximize the rate of backscatter link provided the cellular user’s link quality can be guaranteed.

Let $R_{1}$ be the targeted data rate for cellular link. In the first phase of relaying transmission, the successful decoding set, denoted as $D_{l}$, is defined as $D_{l}=\left\{r_{k}:\min\left\{\gamma_{sr_{k}},\gamma_{r_{k}d}\right\}\geq \gamma_{th_{1}},r_{k}\in R\right\}$, where $\gamma_{th_{1}}=2^{R_{1}}-1$ is the outage threshold SINR for cellular link and the cardinality of $D_{l}$ is *l*. For notational convenience, let

(6) $$w_{r_{k}}=\min\left\{\gamma_{sr_{k}},\gamma_{r_{k}d}\right\}$$

Then, the probability of $D_{l}$ is given by

(7) $$\mathrm{Pr}\left[D_{l}\right]=\mathrm{Pr}\left[\underset{r_{k}\in D_{l}}{⋂}w_{r_{k}}\geq \gamma_{th_{1}},\underset{r_{k}\notin D_{l}}{⋂}w_{r_{k}}<\gamma_{th_{1}}\right]$$

In the second phase of relaying transmission, the relay which has the maximum $\gamma_{r_{k}b}$, denoted as $r_{k^{*}}$, is selected from $D_{l}$ to forward the signal, i.e.,

(8) $$r_{k^{*}}=\arg\max_{r_{k}\in D_{l}}\gamma_{r_{k}b}$$

### 3.2. Proactive RSS

Unlike the conventional proactive RSS where only a type of link is considered, this paper involves two types of links, i.e., cellular link and backscatter link. To employ the conventional proactive RSS, we can regard the backscatter link as a virtual hop (the third hop) in the cellular system. Another problem is that different targeted data rates for cellular link and backscatter link are assumed in this paper, while the same targeted data rate at each hop is assumed in the conventional proactive RSS. We resort to a scale method to tackle this problem. Let $R_{2}$ be the targeted data rate for backscatter link. The proposed proactive RSS is given as follows

(9) $$r_{k^{*}}=\arg\max_{r_{k}\in R}\min\left\{\gamma_{sr_{k}},\gamma_{r_{k}d},\frac{\gamma_{{th}_{1}}}{\gamma_{{th}_{2}}}\gamma_{r_{k}b}\right\}$$

where $\gamma_{th_{2}}=2^{R_{2}}-1$ is the outage threshold SINR for backscatter link. By this method, the same outage threshold SINR $\gamma_{th_{2}}$ can be used for both links.

### 3.3. Relay Selection Implementation

From (8) and (9), both relay selection schemes rely on the instantaneous channel state information (CSI) or instantaneous SINR of each hop. In this section, we will focus on the processes of how to achieve the instantaneous CSI or SINR and how to implement the relay selection in a distributed manner [31]. We assume that all the channels obey reciprocity and keep unchanged during the relay selection procedure and the data communication.

#### 3.3.1. The Reactive Relay Selection Scheme

The steps are given as follows [35]:

(a) The base station transmits a information signal to all relays. All relays try to decode the signal. The relays that successfully decode the signal join the decoding set A.

(b) The destination broadcasts a short pilot signal at a rate $R_{1}=\log_{2}\left(1+\gamma_{th_{1}}\right)$ to backscatter device and all relays. the relays in A that can decode the pilot signal correctly form the active set $D_{l}$. All relays also estimate $\gamma_{r_{k}b}$, $r_{k}\in R$ by using the channel estimation method proposed in [39,40].

(c) If $D_{l}=⌀$, then no best relay will be selected and the system declares an outage event. Otherwise, the relays in $D_{l}$ starts a timer $T_{k}$ and remains silent for the duration inversely proportional to $\gamma_{r_{k}b}$.

(d) The relay whose timer expires first will broadcast a flag packet to the other relays, indicating that they can keep silent for the rest of the current transmission period.

#### 3.3.2. The Proactive Relay Selection Scheme

The steps are given as follows [36]:

(a) The base station transmits a short pilot signal to all relays. All relays estimate the CSI and the instantaneous SINR from the source to themselves, i.e., $g_{sr_{k}}$ and $\gamma_{sr_{k}}$, $r_{k}\in R$.

(b) The destination broadcasts a pilot signal to backscatter device and all relays. All relays estimate the instantaneous CSI and SINR, i.e., $g_{r_{k}d}$, $g_{r_{k}b}g_{bd}$, $\gamma_{r_{k}d}$, and $\gamma_{r_{k}b}$, $r_{k}\in R$, by using the channel estimation method proposed in [39,40].

(c) Each relay $r_{k}$ starts a timer $T_{k}$ and remains silent for the duration inversely proportional to $\min\{\gamma_{sr_{k}},\gamma_{r_{k}d},\frac{\gamma_{{th}_{1}}}{\gamma_{{th}_{2}}}\gamma_{r_{k}b}\}$.

(d) The relay whose timer expires first will broadcast a flag packet to the other relays, indicating that they can keep silent for the rest of the current transmission period.

## 4. Performance Analysis

In this section, we analyze the OP for both RSSs. The OP comparison between different schemes is also provided to gain a better understanding of the property of the proposed schemes.

### 4.1. Reactive RSS

#### 4.1.1. OP Analysis for Backscatter Link

By invoking the total probability law, the OP can be expressed as [31]

(10) $$P_{\mathrm{out},bl}^{\mathrm{react}}=\sum_{l=0}^{K}\sum_{D_{l}}\underset{I_{1}\left(l\right)}{\underset{︸}{\mathrm{Pr}\left[\max_{r_{k}\in D_{l}}\gamma_{r_{k}b}<\gamma_{th_{2}},D_{l}\right]}}$$

where $I_{1}\left(l\right)$ denotes the OP given the cardinality of $D_{l}$.

For notational convenience, let $X=g_{bd}$, $Y=g_{r_{k}b}$, $Z_{k}=\min\left\{\gamma_{1}g_{sr_{k}},\frac{\gamma_{2}g_{r_{k}d}}{\alpha^{2}\gamma_{2}yx+1}\right\}$ with given *x* and *y*, and $V_{k}=\min\left\{\gamma_{1}g_{sr_{k}},\frac{\gamma_{2}g_{r_{k}d}}{\alpha^{2}\gamma_{2}g_{r_{k}b}x+1}\right\}$ with given *x*. Then, the PDFs of *X* and *Y* can be expressed as

(11) $$\begin{array}{cc} f_{X}\left(x\right) & =\lambda_{bd}\exp\left(-\lambda_{bd}x\right),\;x\geq 0\\ f_{Y}\left(y\right) & =\lambda_{rb}\exp\left(-\lambda_{rb}y\right),\;y\geq 0 \end{array}$$

According to [41], the cumulative distribution function (CDF) of $Z_{k}$ and $V_{k}$ can be obtained as

(12) $$\begin{array}{cc} F_{Z_{k}}\left(z\right) & =1-\exp\left(-(\frac{\lambda_{sr}}{\gamma_{1}}+\frac{\lambda_{rd}}{\gamma_{2}}+\alpha^{2}\lambda_{rd}xy)z\right) \end{array}$$

(13) $$\begin{array}{cc} F_{V_{k}}\left(v\right) & =1-\frac{\lambda_{rb}}{\alpha^{2}\lambda_{rd}xv+\lambda_{rb}}\exp\left(-(\frac{\lambda_{sr}}{\gamma_{1}}+\frac{\lambda_{rd}}{\gamma_{2}})v\right) \end{array}$$

By substituting (7) into (10) and considering the correlation among $\gamma_{r_{k}d}$, $\gamma_{r_{m}d}$, and $\gamma_{r_{k}b}$, ($k\neq m,r_{k},r_{m}\in R$), $I_{1}\left(l\right)$ can be rewritten as

(14) $$\begin{array}{cc} I_{1}\left(l\right) & =\mathrm{Pr}[\underset{r_{k}\in D_{l}}{⋂}\left(\alpha^{2}\gamma_{2}g_{r_{k}b}g_{bd}<\gamma_{th_{2}}\right),\underset{r_{k}\in D_{l}}{⋂}\left(\min\left\{\gamma_{1}g_{sr_{k}},\frac{\gamma_{2}g_{r_{k}d}}{\alpha^{2}\gamma_{2}g_{r_{k}b}g_{bd}+1}\right\}\geq \gamma_{th_{1}}\right),\\ \; & \;\;\underset{r_{k}\notin D_{l}}{⋂}\left(\min\left\{\gamma_{1}g_{sr_{k}},\frac{\gamma_{2}g_{r_{k}d}}{\alpha^{2}\gamma_{2}g_{r_{k}b}g_{bd}+1}\right\}<\gamma_{th_{1}}\right)]\\ \; & =\int_{0}^{\infty }\underset{I_{2}}{\underset{︸}{\prod_{r_{k}\in D_{l}}\left\{\int_{0}^{\frac{\gamma_{th_{2}}}{\alpha^{2}\gamma_{2}x}}\mathrm{Pr}\left[Z_{k}\geq \gamma_{th_{1}}\right]f_{Y}\left(y\right)dy\right\}}}\underset{I_{3}}{\underset{︸}{\prod_{r_{k}\notin D_{l}}\mathrm{Pr}\left[V_{k}<\gamma_{th_{1}}\right]}}f_{X}\left(x\right)dx \end{array}$$

where the derivation of (14) can be found in Appendix A for brevity.

Substituting the CDF of $Z_{k}$ into (14) leads to $I_{2}$ given by

(15) $$\begin{array}{c} I_{2}={\left[\int_{0}^{\frac{\gamma_{th_{2}}}{\alpha^{2}\gamma_{2}x}}\lambda_{rb}\exp\left(-\left(\frac{\lambda_{sr}}{\gamma_{1}}+\frac{\lambda_{rd}}{\gamma_{2}}+\alpha^{2}\lambda_{rd}xy\right)\gamma_{th_{1}}-\lambda_{rb}y\right)dy\right]}^{l} \end{array}$$

Performing integration with respect to *y*, we have

(16) I2=ax+al∑i=0lli(−1)iexp−(1a+1x)b·i−c·l

where $a=\frac{\lambda_{rb}}{\alpha^{2}\lambda_{rd}\gamma_{th_{1}}}$, $b=\frac{\lambda_{rb}\gamma_{th_{2}}}{\alpha^{2}\gamma_{2}}$, and $c=\left(\frac{\lambda_{sr}}{\gamma_{1}}+\frac{\lambda_{rd}}{\gamma_{2}}\right)\gamma_{th_{1}}$. By substituting (12) into $I_{3}$, $I_{3}$ is obtained as

(17) I3=∑j=0K−lK−lj(−1)jax+ajexp−c·j

By substituting (16) and (17) into (14), we have

(18) I1(l)=∑i=0l∑j=0K−lliK−lj(−1)i+jexp−c(l+j)−bai·I4

where

(19) $$\begin{array}{c} I_{4}=\int_{0}^{\infty }\lambda_{bd}{\left(\frac{a}{x+a}\right)}^{l+j}\exp\left(-\lambda_{bd}x-b\cdot i/x\right)dx \end{array}$$

Unfortunately, it is difficult, if not impossible, to obtain the exact closed-form expression for $I_{4}$. Thus, we use Gauss–Chebyshev quadrature to approximate $I_{4}$ as follows:

(20) $$I_{4}\approx \frac{\lambda_{bd}\pi \Omega_{1}}{2N}\sum_{n=1}^{N}\sqrt{1-\psi_{n}^{2}}{\left(\frac{a}{\nu_{n}+a}\right)}^{l+j}\exp\left(-\lambda_{bd}\nu_{n}-\frac{b}{\nu_{n}}i\right)$$

where *N* is a complexity-versus-accuracy tradeoff parameter, $\Omega_{1}$ is a large value, $\psi_{n}=\cos\frac{(2n-1)\pi }{2N}$, and $\nu_{n}=\left(\psi_{n}+1\right)\Omega_{1}/2$. Note that an acceptable accuracy can be achieved for a small value of *N* and a appropriately large value of $\Omega_{1}$, i.e., N=40, $\Omega_{1}=500$, which is verified in simulations.

Combining (10), (18) and (20), we have the following theorem.

**Theorem** **1.** *The OP of backscatter link for the reactive RSS is given by*

(21) Pout,blreact≈λbdπΩ12N∑l=0K∑i=0l∑j=0K−l∑n=1NKlliK−lj(−1)i+j·1−ψn2aνn+al+jexp−(1a+1νn)b·i−c(l+j)−λbdνn

#### 4.1.2. OP Analysis for Cellular Link

For the reactive RSS, if no relay joins $D_{l}$, i.e., $D_{l}=⌀$, an outage event occurs. Thus, the OP of cellular link for the reactive RSS can be expressed as

(22) $$\begin{array}{c} P_{\mathrm{out},cl}^{\mathrm{react}}=\mathrm{Pr}\left[\max_{r_{k}\in R}\min\left\{\gamma_{sr_{k}},\gamma_{r_{k}d}\right\}<\gamma_{th_{1}}\right] \end{array}$$

Considering the correlation among $\gamma_{r_{k}d}$ and $\gamma_{r_{m}d}$, ($k\neq m,r_{k},r_{m}\in R$), (22) can be rewritten as

(23) $$\begin{array}{c} P_{\mathrm{out},cl}^{\mathrm{react}}=\int_{0}^{\infty }\prod_{r_{k}\in R}\mathrm{Pr}\left[V_{k}<\gamma_{th_{1}}\right]f_{X}\left(x\right)dx \end{array}$$

Substituting the CDF of $V_{k}$ into (23), and after some straightforward steps, we have

(24) Pout,clreact=∑i=0KKi(−1)iexp−c·i∫0∞ax+aiλbdexp−λbdxdx

With the aid of (Equation (3.462.15) [42]), the OP is obtained as

(25) Pout,clreact=∑i=0KKi(−λbda)iexp−c·i+λbdaΓ(1−i,λbda)

where Γ(·,·) is the upper incomplete gamma function (Equation (8.350.2) [42]).

### 4.2. Proactive RSS

#### 4.2.1. OP Analysis for Backscatter Link

From (9), the OP for the proactive RSS is expressed as

(26) $$P_{\mathrm{out},bl}^{\mathrm{proact}}=\mathrm{Pr}\left[\max_{r_{k}\in R}\min\left\{\gamma_{sr_{k}},\gamma_{r_{k}d},\frac{\gamma_{{th}_{1}}}{\gamma_{{th}_{2}}}\gamma_{r_{k}b}\right\}<\gamma_{th_{1}}\right]$$

By substituting (2), (4) and (5) into (26), and after some straightforward steps, the OP can be expressed as

(27) $$\begin{array}{c} P_{\mathrm{out},bl}^{\mathrm{proact}}=\int_{0}^{\infty }\prod_{r_{k}\in R}\left\{1-\int_{\frac{\gamma_{th_{2}}}{\alpha^{2}\gamma_{2}x}}^{\infty }\mathrm{Pr}\left[Z_{k}<\gamma_{th_{1}}\right]f_{Y}\left(y\right)dy\right\}f_{X}\left(x\right)dx \end{array}$$

where the derivation of (27) can be found in Appendix B for brevity.

Substituting the CDF of $Z_{k}$ into (27) and performing integration with respect to *y*, we have

(28) $$\begin{array}{c} P_{\mathrm{out},bl}^{\mathrm{proact}}=\int_{0}^{\infty }{\left[1-\frac{a}{x+a}\exp\left(-\frac{b}{a}-\frac{b}{x}-c\right)\right]}^{K}\lambda_{bd}\exp\left(-\lambda_{bd}x\right)dx \end{array}$$

By invoking the binomial theorem and with the aid of Gauss–Chebyshev quadrature, the OP for the proactive RSS can be given in the following theorem.

**Theorem** **2.** *The OP of backscatter link for the proactive RSS is given by*

(29) Pout,blproact≈λbdπΩ22M∑i=0K∑m=1MKi(−1)i1−ϕm2·aξm+aiexp−(1a+1ξm)b·i−c·i−λbdξm

*where M is a complexity-versus-accuracy tradeoff parameter, $\Omega_{2}$ is a large value, $\phi_{m}=\cos\frac{(2m-1)\pi }{2M}$, and $\xi_{m}=\left(\phi_{m}+1\right)\Omega_{2}/2$.*

**Remark** **1.** *Impact of the number of relays: By taking the partial derivative of (28) with respect to K, we have*

(30) $$\begin{array}{cc} \frac{\partial P_{out,bl}^{proact}}{\partial K} & =\int_{0}^{\infty }{\left[1-\frac{a}{x+a}\exp\left(-\frac{b}{a}-\frac{b}{x}-c\right)\right]}^{K}\lambda_{bd}\exp\left(-\lambda_{bd}x\right)\\ \; & \cdot \ln\left[1-\frac{a}{x+a}\exp\left(-\frac{b}{a}-\frac{b}{x}-c\right)\right]dx<0 \end{array}$$

*Thus, the OP can be improved via increasing K.*

#### 4.2.2. OP Analysis for Cellular Link

For notational convenience, let

(31) $$\eta_{r_{k}}=\min\left\{\gamma_{sr_{k}},\gamma_{r_{k}d},\frac{\gamma_{{th}_{1}}}{\gamma_{{th}_{2}}}\gamma_{r_{k}b}\right\}$$

Then, the OP of cellular link for the proactive RSS can be expressed as

(32) $$\begin{array}{c} P_{\mathrm{out},cl}^{\mathrm{proact}}=\sum_{k=1}^{K}\mathrm{Pr}\left[w_{r_{k}}<\gamma_{th_{1}},\eta_{r_{k}}>\max_{r_{i}\in R,r_{i}\neq r_{k}}\eta_{r_{i}}\right] \end{array}$$

Unfortunately, there is no closed-form expression for (32) due to the complicated correlation among $\gamma_{r_{k}d}$, $\gamma_{r_{m}d}$, and $\gamma_{r_{k}b}$ ($k\neq m,r_{k},r_{m}\in R$).

### 4.3. Performance Comparison

For the backscatter link, we have the following corollary.

**Corollary** **1.** *For the backscatter link, the OP relationship between the reactive RSS and proactive RSS at all SNR regimes is*

(33) $$P_{out,bl}^{react}=P_{out,bl}^{proact}$$

**Proof** **of** **Corollary** **1.** Reformulate $I_{3}$ in (14) as follows:

(34) $$\begin{array}{cc} I_{3} & =\prod_{r_{k}\notin D_{l}}\left\{1-\mathrm{Pr}\left[V_{k}\geq \gamma_{th_{1}}\right]\right\}\\ \; & =\prod_{r_{k}\notin D_{l}}\left\{1-\int_{0}^{\infty }\mathrm{Pr}\left[Z_{k}\geq \gamma_{th_{1}}\right]f_{Y}\left(y\right)dy\right\} \end{array}$$

Substituting (14) and (34) into (10), we have

(35) $$\begin{array}{cc} \; & P_{\mathrm{out},bl}^{\mathrm{react}}=\sum_{l=0}^{K}\sum_{D_{l}}\int_{0}^{\infty }\prod_{r_{k}\in D_{l}}\left\{\int_{0}^{\frac{\gamma_{th_{2}}}{\alpha^{2}\gamma_{2}x}}\mathrm{Pr}\left[Z_{k}\geq \gamma_{th_{1}}\right]f_{Y}\left(y\right)dy\right\}\\ \; & \cdot \prod_{r_{k}\notin D_{l}}\left\{1-\int_{0}^{\infty }\mathrm{Pr}\left[Z_{k}\geq \gamma_{th_{1}}\right]f_{Y}\left(y\right)dy\right\}f_{X}\left(x\right)dx \end{array}$$

By invoking the binomial theorem, (35) can be rewritten as

(36) $$\begin{array}{c} P_{\mathrm{out},bl}^{\mathrm{react}}=\int_{0}^{\infty }\prod_{r_{k}\in R}\left\{1-\int_{\frac{\gamma_{th_{2}}}{\alpha^{2}\gamma_{2}x}}^{\infty }\mathrm{Pr}\left[Z_{k}<\gamma_{th_{1}}\right]f_{Y}\left(y\right)dy\right\}f_{X}\left(x\right)dx \end{array}$$

which is identical to (27), and the final result is obtained. □

For the cellular link, the following corollary can be obtained.

**Corollary** **2.** *For the cellular link, the OP relationship between the reactive RSS and proactive RSS at all SNR regimes is*

(37) $$P_{out,cl}^{react}\leq P_{out,cl}^{proact}$$

**Proof** **of** **Corollary** **2.** Note that $\eta_{r_{k}}=\min\left\{w_{r_{k}},\frac{\gamma_{{th}_{1}}}{\gamma_{{th}_{2}}}\gamma_{r_{k}b}\right\}$. Now, relaying on the relation between $w_{r_{k}}$ and $\gamma_{{th}_{1}}\gamma_{r_{k}b}/\gamma_{{th}_{2}}$, we define the set

(38) $$B_{n}=\left\{r_{k}:w_{r_{k}}<\frac{\gamma_{{th}_{1}}}{\gamma_{{th}_{2}}}\gamma_{r_{k}b},r_{k}\in R\right\},$$

where the cardinality of $B_{n}$ is *n*. The OP for the proactive RRS (32) can be rewritten as

(39) $$\begin{array}{cc} P_{\mathrm{out},cl}^{\mathrm{proact}} & =\sum_{k=1}^{K}\mathrm{Pr}\left[w_{r_{k}}<\gamma_{th_{1}},w_{r_{k}}>\max_{r_{i}\in R,r_{i}\neq r_{k}}w_{r_{i}}\right]\\ \; & +\sum_{k=1}^{K}\sum_{n=1}^{K}\sum_{B_{n}}\mathrm{Pr}\left[w_{r_{k}}<\gamma_{th_{1}},\eta_{r_{k}}>\max_{r_{i}\in R,r_{i}\neq r_{k}}\eta_{r_{i}},B_{n}\right] \end{array}$$

where the first and second terms correspond to the cases where n=0 and n>0, respectively. Note that the first term in (39) is identical to $P_{\mathrm{out},cl}^{\mathrm{react}}$ and the second term is a non-negative number. The final result is obtained. □

About the OP relationship between the backscatter link and cellular link, we have the following corollary.

**Corollary** **3.** *The OP relationship of the backscatter link and cellular link at all SNR regimes is*

(40) $$P_{out,cl}^{react}\leq P_{out,cl}^{proact}\leq P_{out,bl}^{proact}=P_{out,bl}^{react}$$

**Proof** **of** **Corollary** **3.** Since $\eta_{r_{k}}\leq w_{r_{k}}$, we have

(41) $$\begin{array}{c} P_{\mathrm{out},cl}^{\mathrm{proact}}\leq \sum_{k=1}^{K}\mathrm{Pr}\left[\eta_{r_{k}}<\gamma_{th_{1}},\eta_{r_{k}}>\max_{r_{i}\in R,r_{i}\neq r_{k}}\eta_{r_{i}}\right]=P_{\mathrm{out},bl}^{\mathrm{proact}} \end{array}$$

By combing (33) and (37), the final result is obtained. □

The following theorem shows the optimality of the proposed reactive RSS.

**Theorem** **3.** *For the cooperative AmBC system, the reactive RSS is outage-optimal among single-relay selection schemes, which means the minimum OP can be achieved simultaneously for both cellular and backscatter links.*

**Proof** **of** **Theorem** **3.** This can be proved by contradiction. Due to the SIC technique employed at *d*, the performance of the cellular link should be guaranteed first. According to whether the relays can decode the signal from *s* or not, two cases should be considered. (1) In case I, no relay can decode $x_{s}$ correctly, i.e., $D_{l}=⌀$, and an outage event occurs for both cellular and backscatter links. It is meaningless to compare the performance among different relay selection schemes in this case. (2) In case II, $D_{l}\neq ⌀$, there is at least one relay that can decode $x_{s}$ successfully. In this case, the outage performance of the cellular link can be guaranteed. The performance of a RSS only depends on the outage performance of the backscatter link. Assume that there exists a better RSS that can achieve a lower OP for the backscatter link than the OP of the reactive RSS. In other words, there exists a relay $r_{k^{\circ }}\in D_{l}$, $r_{k^{\circ }}\neq r_{k^{*}}$, selected by the better RSS, and an outage event does not occur for $r_{k^{\circ }}$-*b*-*d* link, while it occurs for $r_{k^{*}}$-*b*-*d* link, which implies the link quality of $r_{k^{\circ }}$-*b*-*d* is stronger than the link quality of $r_{k^{*}}$-*b*-*d*, i.e., $\gamma_{r_{k}^{\circ }b}>\gamma_{r_{k}^{*}b}$. This is impossible because it is contrary to the fact $r_{k^{*}}=\arg\max_{r_{k}\in D_{l}}\gamma_{r_{k}b}$. Hence, the reactive RSS is outage-optimal among single-relay selection schemes. □

## 5. Asymptotic Performance Analysis

In this section, simple expressions are derived at high SNR to gain some insights for system design. Specifically, we have the following corollary.

**Corollary** **4.** *Let $\gamma_{2}=\mu \gamma_{1}$, where μ is a constant. When $\gamma_{1}\to \infty$, the asymptotic OPs of cellular link and backscatter link for both RSSs at high SNR regime are*

(42) Pout,χψ≈γ1→∞∑i=0KKi(−λbda)iexpλbdaΓ(1−i,λbda)

*where ψ∈{react,proact},χ∈{bl,cl}.*

**Proof** **of** **Corollary** **4.** Let $\gamma_{2}=\mu \gamma_{1}$, where μ is a constant.

(1) For the backscatter link of the proactive RSS, when $\gamma_{1}\to \infty$, by using the Taylor approximation, exp(x)≈1+x, $P_{\mathrm{out},bl}^{\mathrm{proact}}$ in (28) can be approximated as

(43) $$\begin{array}{c} P_{\mathrm{out},bl}^{\mathrm{proact}}\approx \int_{0}^{\infty }{\left[1-\frac{a}{x+a}\left(1-\frac{b}{a}-\frac{b}{x}-c\right)\right]}^{K}\lambda_{bd}\exp\left(-\lambda_{bd}x\right)dx \end{array}$$

By using the binomial theorem and neglecting the term related to $1/\gamma_{1}$, we have

(44) Pout,blproact≈∫0∞∑i=0KKi(−1)iλbdai(x+a)iexp−λbdxdx

With the aid of (Equation (3.462.15) [42]), $P_{\mathrm{out},bl}^{\mathrm{proact}}$ can be approximated as

(45) Pout,blproact≈∑i=0KKi(−λbda)iexpλbdaΓ(1−i,λbda)

According to Corollary 1, the asymptotic OP for the reactive RSS is identical to (45).

(2) For the cellular link of the reactive RSS, when $\gamma_{1}\to \infty$, by using the Taylor approximation in (25), we have

(46) Pout,clreact≈∑i=0KKi(−λbda)iexpλbdaΓ(1−i,λbda)

Note that the expression of (46) is the same as (45). Then, by combing (40), (45) and (46) and invoking the squeeze theorem, the asymptotic OP of the cellular link for the proactive RSS can be expressed as

(47) Pout,clproact≈∑i=0KKi(−λbda)iexpλbdaΓ(1−i,λbda)

Finally, combing (45)–(47), the final result is obtained. □

**Remark** **2.** *Diversity Order: According to the definition of diversity order, we have $G_{d}=-\lim_{\gamma_{1}\to \infty }\frac{\log P_{out,\chi }^{\psi }\left(\gamma_{1}\right)}{\log(\gamma_{1})}=0$. Interesting, an outage floor occurs at high SNR, i.e., the OP at high SNR is independent of $\gamma_{1}$ and $\gamma_{2}$.*

**Remark** **3.** *Corollary 4 shows that the same outage floor is achieved for both RSSs when $\gamma_{2}=\mu \gamma_{1}$ and $\gamma_{1}\to \infty$.*

**Remark** **4.** *Impact of the reflection coefficient: By taking the partial derivative of (42) with respect to α, we can obtain*

(48) $$\begin{array}{cc} \frac{\partial P_{out,\chi }^{\psi }}{\partial \alpha } & =\frac{2K\lambda_{bd}}{a\alpha }\int_{0}^{\infty }\frac{x^{K}}{{\left(x+a\right)}^{K+1}}\exp\left(-\lambda_{bd}x\right)dx>0 \end{array}$$

*This means the outage floor can be reduced with the decreasing of the reflection coefficient.*

## 6. Simulation Results

**Figure 2:**
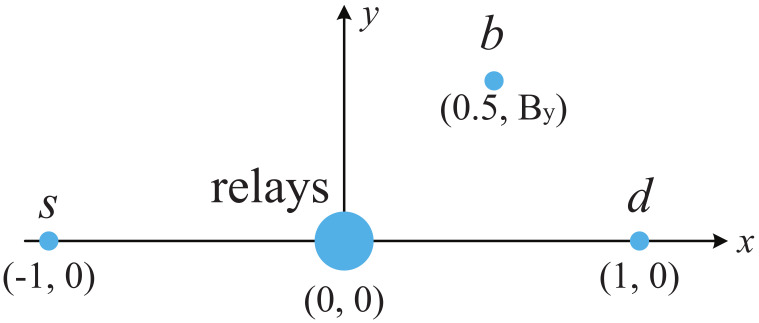
The positions of base station (s), relays, backscatter device (b), and destination (d) in the simulations.

In this section, we present Monte Carlo simulation results to verify our analysis. The simulation tool is MATLAB. In the simulations, as shown in Figure 2, a two dimensional network topology is assumed where base station (s), the relays, the backscatter device (b), and the destination (d) are located at the coordinates (−1,0), (0,0), $\left(0.5,B_{y}\right)$, and (1,0), respectively [18,29,30]. The fading variances are assigned by adopting a path loss model of the form $\lambda_{ij}=d_{ij}^{-\beta }$ where $d_{ij}$ is the distance between the transmitter node *i* (*s*, $r_{1}$, ⋯, $r_{K}$, *b*) and receiver node *j* ($r_{1}$, ⋯, $r_{K}$, *b*, *d*), and β denotes the path loss factor. We assume that all the relays are located close to one another (optimal clustering) [37,38]. The distance between any two relays is negligible compared with that between the relays and the nodes *s*, *b*, and *d*. Thus, we have $\lambda_{sr_{k}}=\lambda_{sr}$, $\lambda_{r_{k}d}=\lambda_{rd}$, and $\lambda_{r_{k}b}=\lambda_{rb}$, $r_{k}\in R$. Unless otherwise specified, we set $B_{y}=0.6$, α=0.3, β=2, $\gamma_{1}=\gamma_{2}=20$ dB, K=8, M=N=40, and $\Omega_{1}=\Omega_{2}=500$. In all cases, $R_{1}=1$ bps/Hz and $R_{2}=0.3$ bps/Hz.

**Figure 3:**
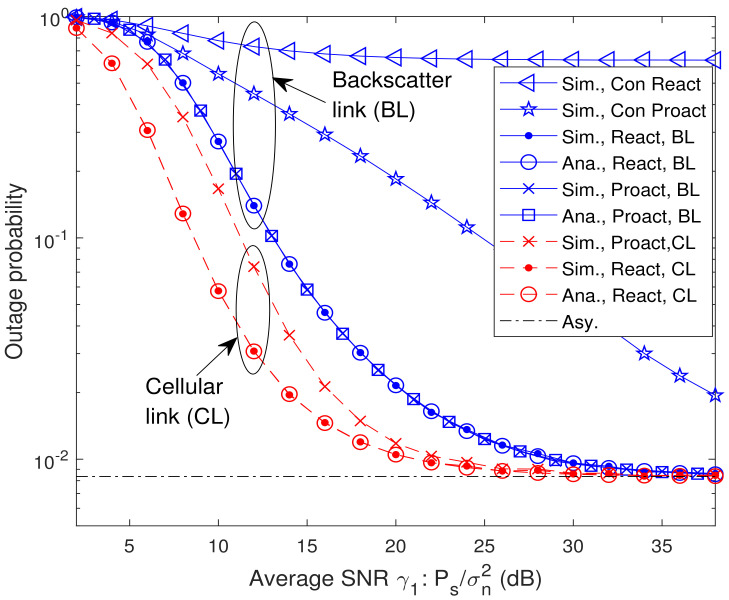
Analytical and simulated OP versus average SNR for different relay selection schemes with K=8 and $\mu =\gamma_{2}/\gamma_{1}=1$.

In Figure 3, we present the analytical and simulated OP versus average SNR for different relay selection schemes. In the legend, “Sim.”, “Ana.”, and “Asy.” denote the simulated results, the analytical results and the asymptotic results, respectively, and “React, BL”, “Proact, BL”, “React, CL”, and “Proact, CL” denote the OP of backscatter link for the reactive RSS (21), the OP of backscatter link for the proactive RSS (29), the OP of cellular link for the reactive RSS (25), and the OP of cellular link for the proactive RSS (32), respectively. Additionally, the labels “Con React” and “Con Proact” in these figures stand for the OP of backscatter link for the conventional proactive RSS, i.e., $r_{k^{*}}=\arg\max_{r_{k}\in R}\min\left\{\gamma_{sr_{k}},\gamma_{r_{k}d}\right\}$, and conventional reactive RSS, i.e., $r_{k^{*}}=\arg\max_{r_{k}\in A_{l}}\gamma_{r_{k}d}$, where $A_{l}=\left\{r_{k}\in R,\gamma_{sr_{k}}\geq \gamma_{th_{1}}\right\}$, respectively [31,32]. From Figure 3, it is observed that the analytical results match the simulation ones well. As expected, the OPs of backscatter link for both reactive RSS and proactive RSS are identical as indicated in Lemma 1. In addition, when $\mu =\gamma_{2}/\gamma_{1}=1$, the OPs of backscatter link for both RSSs decrease with the increase of average SNR $\gamma_{1}$. At high SNR, the outage floor, whose value identical to the asymptotic OP at high SNR (42), occurs. The outage floor phenomenon can be interpreted as follows. From (9), the OPs of backscatter link for both RSSs depend on $\min\{\gamma_{sr_{k}},\gamma_{r_{k}d},\frac{\gamma_{{th}_{1}}}{\gamma_{{th}_{2}}}\gamma_{r_{k}b}\}$. According to (2)–(5), $\gamma_{sr_{k}}$, $\gamma_{r_{k}d}$, and $\gamma_{r_{k}b}$ are increasing functions of $\gamma_{1}$ or $\gamma_{2}$. Since μ is fixed, i.e., $\gamma_{1}$ is proportional to $\gamma_{2}$, the OP decreases with the increase of $\gamma_{1}$ at lower SNR region. However, when $\gamma_{1}$ increases further, $\gamma_{r_{k}d}$ tends to be stable in (4) which causes outage floor. Results also show that the reactive RSS is outage-optimal compared with the other single-relay selection schemes, which is consistent with Theorem 3.

**Figure 4:**
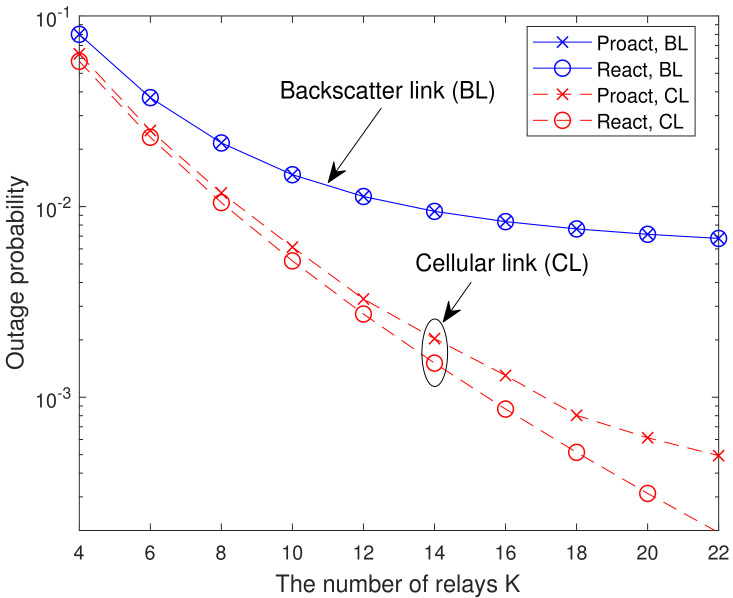
Analytical and simulated OP versus the number of relays *K* with $\gamma_{1}=\gamma_{2}=20$ dB.

In Figure 4, we present the analytical and simulated OP versus the number of relays *K* with $\gamma_{1}=\gamma_{2}=20$ dB. As shown in Figure 4, the OPs of the backscatter link and cellular link for both RSSs decrease with the increase of *K*. The reason is that the more relays are available, the higher end-to-end SINR can be achieved. Thus, the outage floor can be reduced by increasing the number of relays.

**Figure 5:**
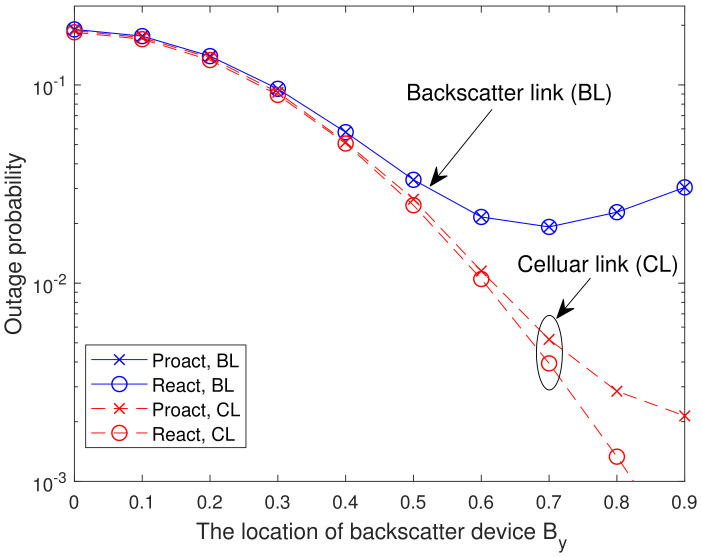
Analytical and simulated OP versus the location of backscatter device $B_{y}$ with $\gamma_{1}=\gamma_{2}=20$ dB and K=8.

In Figure 5, we present the analytical and simulated OP versus the location of backscatter device $B_{y}$ with $\gamma_{1}=\gamma_{2}=20$ dB and K=8. For the backscatter link, it can be seen that the minimum OP is attained when $B_{y}=0.7$. This can be interpreted as follows. From (9), the OPs of the backscatter link for both RSSs depend on $\min\{\gamma_{sr_{k}},\gamma_{r_{k}d},\frac{\gamma_{{th}_{1}}}{\gamma_{{th}_{2}}}\gamma_{r_{k}b}\}$. By changing $B_{y}$ from 0 to 0.9, the dyadic backscatter channel quality, i.e., $g_{r_{k}b}g_{bd}$, becomes weaker and weaker, which in turn results in a larger $\gamma_{r_{k}d}$ and a smaller $\gamma_{r_{k}b}$. When $B_{y}$ increases from 0 to 0.7, i.e., $\gamma_{r_{k}d}<\gamma_{{th}_{1}}\gamma_{r_{k}b}/\gamma_{{th}_{2}}$, the OP is limited by $\gamma_{r_{k}d}$ and the OP decreases with the increasing of $B_{y}$. However, when $B_{y}$ increases further, i.e., $\gamma_{r_{k}d}\geq \gamma_{{th}_{1}}\gamma_{r_{k}b}/\gamma_{{th}_{2}}$, the OP is limited by $\gamma_{r_{k}b}$ and the OP increases with the increasing of $B_{y}$. For the cellular link, it can be seen that the OP reaches to the maximum value at $B_{y}=0$ due to the strongest interference caused by the backscatter link at $B_{y}=0$. This means that the BD cannot be located too close to the cellular users to avoid strong interference, nor can it be located too close to the cellular users to avoid sacrificing its own performance. Thus, for the BD, there is an optimal location where the BD and the cellular users can achieve a win-win situation.

**Figure 6:**
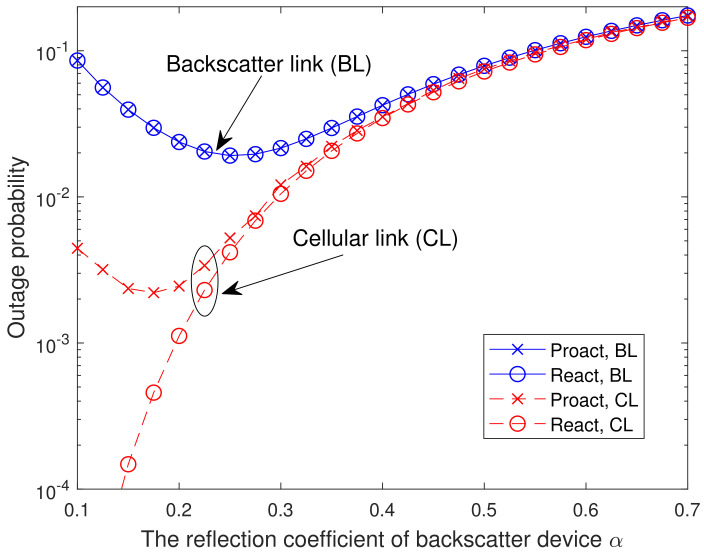
Analytical and simulated OP versus the reflection coefficient of backscatter device α with $\gamma_{1}=\gamma_{2}=20$ dB and K=8.

In Figure 6, we present the analytical and simulated OP versus the reflection coefficient of backscatter device α with $\gamma_{1}=\gamma_{2}=20$ dB and K=8. From Figure 6, it is observed that the OP of backscatter link decreases first and then increases with the increasing of α. The reason is similar to the one provided in Figure 5. Furthermore, the OP of cellular link for the reactive RSS increases as α grows. This can be interpreted as follows. According to (2) and (4), $\gamma_{sr_{k}}$ is independent of α, whereas $\gamma_{r_{k}d}$ decreases with the increase of α. Thus, as α increases, the number of relays in the successful decoding set $D_{l}$ decreases, which leads to the OP deterioration. For the BD, a large reflection factor causes significant interference to the cellular users, but a small reflection factor leads to its own performance degradation. For the reactive RSS, there is an optimal reflection factor that enables both the BD and the cellular users to achieve good performance. However, for the proactive RSS, such an optimal reflection factor does not exist, and a trade-off between the BD and the cellular users is necessary to determine the value of the reflection factor.

**Figure 7:**
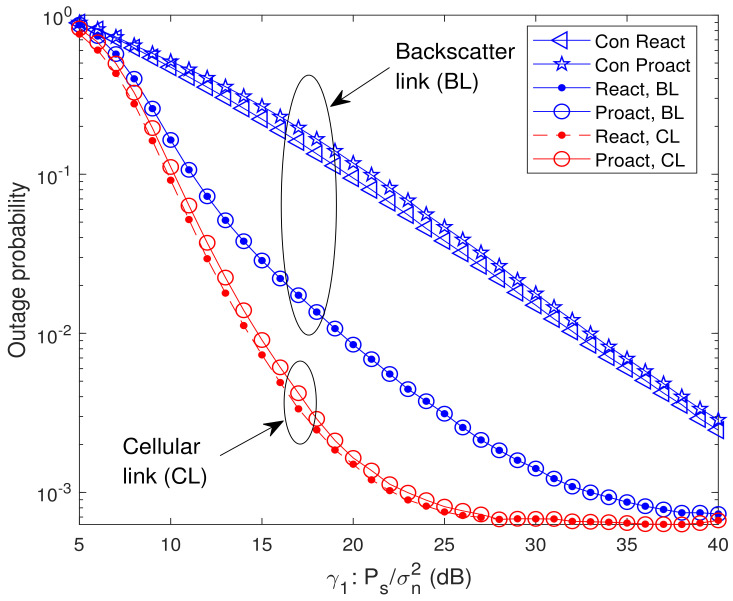
Analytical and simulated OP of the system where the BD is located between *s* and the relays (located at (−0.5,0.6)) with K=16 and $\mu =\gamma_{2}/\gamma_{1}=1$.

**Figure 8:**
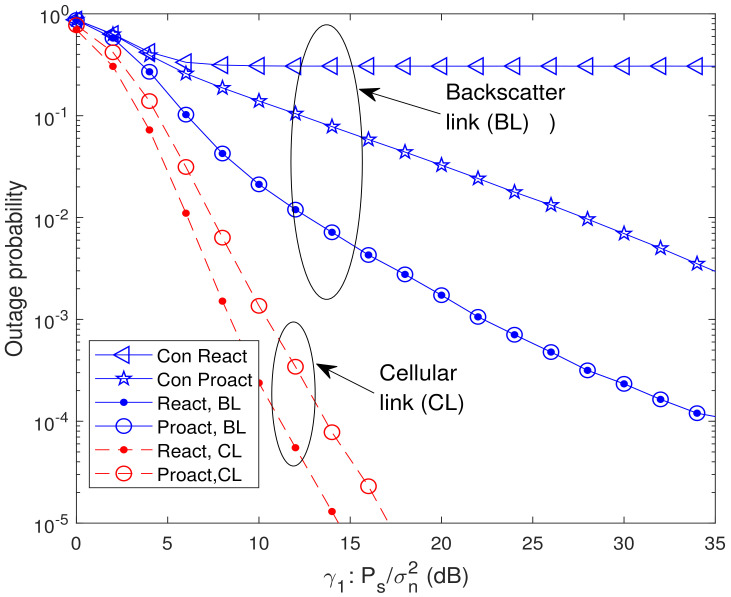
Analytical and simulated OP of the system where the destination *d* is located in the middle (located at (0,0.2)) with K=8, α=0.6, and $\mu =\gamma_{2}/\gamma_{1}=1$.

In Figure 7 and Figure 8, we present the analytical and simulated OP of the systems where the BD is located between *s* and the relays (located at (−0.5,0.6)) and *d* is located in the middle (located at (0,0.2)), respectively. In the former system, for the reactive RSS, $r_{k^{*}}=\arg\max_{r_{k}\in C_{l}}\gamma_{r_{k}db}$, where $C_{l}=\left\{r_{k}\in R,\min\left\{\gamma_{sr_{k}},\gamma_{r_{k}d}\right\}\geq \gamma_{th_{1}},\gamma_{br_{k}}\geq \gamma_{th_{2}}\right\}$ with $\gamma_{sr_{k}}=\frac{\gamma_{1}g_{sr_{k}}}{\alpha^{2}\gamma_{1}g_{sb}g_{br_{k}}+1}$, $\gamma_{br_{k}}=\alpha^{2}\gamma_{1}g_{sb}g_{br_{k}}$, $\gamma_{r_{k}d}=\frac{\gamma_{2}g_{r_{k}d}\rho_{1}^{2}}{\gamma_{2}g_{r_{k}d}\rho_{2}^{2}+1}$, and $\gamma_{r_{k}db}=\gamma_{2}g_{r_{k}d}\rho_{2}^{2}$, and for the proactive RSS, $r_{k^{*}}=\arg\max_{r_{k}\in R}\min\left\{\gamma_{sr_{k}},\gamma_{r_{k}d},\frac{\gamma_{{th}_{1}}}{\gamma_{{th}_{2}}}\gamma_{br_{k}},\frac{\gamma_{{th}_{1}}}{\gamma_{{th}_{2}}}\gamma_{r_{k}db}\right\}$. During the second hop in the former system, the selected relay uses NOMA to forward the backscatter and cellular signals. For the latter system, the RSSs are the same as (8) and (9), and *D* uses maximal ratio combining (MRC) to combine the cellular signals received by the first and second hops. From Figure 7 and Figure 8, it is observed that the OP of the reactive RSS outperforms that of the conventional RSSs, which means our proposed reactive RSS is also available for the extended systems. From Figure 8, it is also observed that the outage floor phenomenon does not occur in the cellular link. The reason is that with the existence of a direct link between *s* and *d*, the received SINR of the cellular link can always increase with the increase of $\gamma_{1}$.

## 7. Conclusions

The study of single-relay selection will be very beneficial for cooperative AmBC system design. In this paper, the outage performance of a dual-hop DF cooperative AmBC system with single-relay selection was studied over i.i.d. Rayleigh fading channels. In particular, the OPs of two proposed RSSs, called reactive RSS and proactive RSS, were derived, which built the relationship between the outage performance and the related system parameters (which include the relay selection scheme, the number of relays, the location of BD, and the reflection coefficient of BD). The theoretical analysis was validated by simulation. Both theoretical analysis and simulation revealed that the reactive RSS is outage-optimal among all possible single-relay selection schemes. The results also demonstrated that an outage floor phenomenon occurs at high SNR due to the interference caused by the backscatter link. By increasing the number of relays or decreasing the reflection coefficient of the backscatter device, the outage floor can be reduced. Perfect channel state information (CSI) was assumed in this paper, and it is important to investigate the impact of the imperfect CSI on the outage performance of dual-hop cooperative AmBC systems with single-relay selection. Another promising future direction is to extend the results of the considered model to that of more general ones, e.g., the system where the BD is located in the middle or the system with full-duplex relays. 

## Data Availability

Not applicable.

## Notations

The following notations are used in this manuscript:

$\mathrm{CN}(u,\sigma^{2})$	Complex Gaussian random variable with mean *u* and variance $\sigma^{2}$
$f_{X}\left(\cdot \right)$	Probability density function (PDF) of *X*
$F_{X}\left(\cdot \right)$	Cumulative distribution function (CDF) of *X*
nk=n!k!(n−k)!	Binomial coefficient
Pr[·]	Probability operator
E[·]	Expectation operator
Γ(·,·)	Upper incomplete gamma function (Equation (8.350.2) [42])
|·|	The absolute value of a complex number
$\frac{\partial f(x)}{\partial x}$	Take the partial derivative of f(x) with respect to *x*

## Appendix A. The Derivation of (14)

By substituting (6) and (7) into (10), $I_{1}\left(l\right)$ can be expressed as

(A1) $$I_{1}\left(l\right)\;\;=\;\;\mathrm{Pr}\left[\max_{r_{k}\in D_{l}}\gamma_{r_{k}b}<\gamma_{th_{2}},\underset{r_{k}\in D_{l}}{⋂}\;\;\min\left\{\gamma_{sr_{k}},\gamma_{r_{k}d}\right\}\geq \gamma_{th_{1}},\underset{r_{k}\notin D_{l}}{⋂}\;\;\min\left\{\gamma_{sr_{k}},\gamma_{r_{k}d}\right\}<\gamma_{th_{1}}\right]$$

Since the term $\left\{\max_{r_{k}\in D_{l}}\gamma_{r_{k}b}<\gamma_{th_{2}}\right\}$ can be reformulated as $\left\{\underset{r_{k}\in D_{l}}{⋂}\gamma_{r_{k}b}<\gamma_{th_{2}}\right\}$, $I_{1}\left(l\right)$ can be rewritten as

(A2) $$I_{1}\left(l\right)\;\;=\;\;\mathrm{Pr}\left[\underset{r_{k}\in D_{l}}{⋂}\gamma_{r_{k}b}<\gamma_{th_{2}},\underset{r_{k}\in D_{l}}{⋂}\;\;\min\left\{\gamma_{sr_{k}},\gamma_{r_{k}d}\right\}\geq \gamma_{th_{1}},\underset{r_{k}\notin D_{l}}{⋂}\;\;\min\left\{\gamma_{sr_{k}},\gamma_{r_{k}d}\right\}<\gamma_{th_{1}}\right]$$

Substituting (2), (4) and (5) into (A2), we have

(A3) $$\begin{array}{cc} I_{1}\left(l\right) & =\mathrm{Pr}[\underset{r_{k}\in D_{l}}{⋂}\left(\alpha^{2}\gamma_{2}g_{r_{k}b}g_{bd}<\gamma_{th_{2}}\right),\underset{r_{k}\in D_{l}}{⋂}\left(\min\left\{\gamma_{1}g_{sr_{k}},\frac{\gamma_{2}g_{r_{k}d}}{\alpha^{2}\gamma_{2}g_{r_{k}b}g_{bd}+1}\right\}\geq \gamma_{th_{1}}\right),\\ \; & \;\;\underset{r_{k}\notin D_{l}}{⋂}\left(\min\left\{\gamma_{1}g_{sr_{k}},\frac{\gamma_{2}g_{r_{k}d}}{\alpha^{2}\gamma_{2}g_{r_{k}b}g_{bd}+1}\right\}<\gamma_{th_{1}}\right)] \end{array}$$

Note that all three terms in the probability operator are dependent due to the common backscatter link $g_{bd}$. Let $X=g_{bd}$. Then, $I_{1}\left(l\right)$ can be expressed as

(A4) $$\begin{array}{cc} I_{1}\left(l\right)=\int_{0}^{\infty } & \mathrm{Pr}\left[\underset{r_{k}\in D_{l}}{⋂}\left(\alpha^{2}\gamma_{2}g_{r_{k}b}x<\gamma_{th_{2}}\right),\underset{r_{k}\in D_{l}}{⋂}\left(\min\left\{\gamma_{1}g_{sr_{k}},\frac{\gamma_{2}g_{r_{k}d}}{\alpha^{2}\gamma_{2}g_{r_{k}b}x+1}\right\}\geq \gamma_{th_{1}}\right)\right]\\ \; & \cdot \mathrm{Pr}\left[\underset{r_{k}\notin D_{l}}{⋂}\left(\min\left\{\gamma_{1}g_{sr_{k}},\frac{\gamma_{2}g_{r_{k}d}}{\alpha^{2}\gamma_{2}g_{r_{k}b}x+1}\right\}<\gamma_{th_{1}}\right)\right]f_{X}\left(x\right)dx \end{array}$$

Let $Y=g_{r_{k}b}$, and considering the correlation between the first term and the second term in the first probability operator, $I_{1}\left(l\right)$ is expressed as

(A5) $$\begin{array}{cc} I_{1}\left(l\right)=\int_{0}^{\infty } & \prod_{r_{k}\in D_{l}}\left\{\int_{0}^{\frac{\gamma_{th_{2}}}{\alpha^{2}\gamma_{2}x}}\mathrm{Pr}\left[\min\left\{\gamma_{1}g_{sr_{k}},\frac{\gamma_{2}g_{r_{k}d}}{\alpha^{2}\gamma_{2}yx+1}\right\}\geq \gamma_{th_{1}}\right]f_{Y}\left(y\right)dy\right\}\\ \; & \cdot \mathrm{Pr}\left[\underset{r_{k}\notin D_{l}}{⋂}\left(\min\left\{\gamma_{1}g_{sr_{k}},\frac{\gamma_{2}g_{r_{k}d}}{\alpha^{2}\gamma_{2}g_{r_{k}b}x+1}\right\}<\gamma_{th_{1}}\right)\right]f_{X}\left(x\right)dx \end{array}$$

For notational convenience, let $Z_{k}=\min\left\{\gamma_{1}g_{sr_{k}},\frac{\gamma_{2}g_{r_{k}d}}{\alpha^{2}\gamma_{2}yx+1}\right\}$ with given *x* and *y*, and $V_{k}=\min\left\{\gamma_{1}g_{sr_{k}},\frac{\gamma_{2}g_{r_{k}d}}{\alpha^{2}\gamma_{2}g_{r_{k}b}x+1}\right\}$ with given *x*. Finally, $I_{1}\left(l\right)$ is obtained as

(A6) $$\begin{array}{c} I_{1}\left(l\right)=\int_{0}^{\infty }\prod_{r_{k}\in D_{l}}\left\{\int_{0}^{\frac{\gamma_{th_{2}}}{\alpha^{2}\gamma_{2}x}}\mathrm{Pr}\left[Z_{k}\geq \gamma_{th_{1}}\right]f_{Y}\left(y\right)dy\right\}\prod_{r_{k}\notin D_{l}}\mathrm{Pr}\left[V_{k}<\gamma_{th_{1}}\right]f_{X}\left(x\right)dx \end{array}$$

Thus, the derivation of (14) is completed.

## Appendix B. The Derivation of (27)

By substituting (2), (4) and (5) into (26), the OP for the proactive RSS is expressed as

(A7) $$P_{\mathrm{out},bl}^{\mathrm{proact}}=\mathrm{Pr}\left[\max_{r_{k}\in R}\underset{\eta_{r_{k}}}{\underset{︸}{\min\left(\gamma_{1}g_{sr_{k}},\frac{\gamma_{2}g_{r_{k}d}}{\alpha^{2}\gamma_{2}g_{r_{k}b}g_{bd}+1},\alpha^{2}\gamma_{2}g_{r_{k}b}g_{bd}\frac{\gamma_{{th}_{1}}}{\gamma_{{th}_{2}}}\right)}}<\gamma_{th_{1}}\right]$$

From (A7), it is noted that $\eta_{r_{k}}$ and $\eta_{r_{m}}$ ($k\neq m,r_{k},r_{m}\in R$) are dependent due to the common random variable $g_{bd}$. Then, (A7) can be expressed as

(A8) $$P_{\mathrm{out},bl}^{\mathrm{proact}}=\int_{0}^{\infty }\mathrm{Pr}\left[\max_{r_{k}\in R}\min\left(\gamma_{1}g_{sr_{k}},\frac{\gamma_{2}g_{r_{k}d}}{\alpha^{2}\gamma_{2}g_{r_{k}b}x+1},\alpha^{2}\gamma_{2}g_{r_{k}b}x\frac{\gamma_{{th}_{1}}}{\gamma_{{th}_{2}}}\right)<\gamma_{th_{1}}\right]f_{g_{bd}}\left(x\right)dx$$

Subsequently, the $P_{\mathrm{out},bl}^{\mathrm{proact}}$ in (A8) can be rewritten as

(A9) $$P_{\mathrm{out},bl}^{\mathrm{proact}}=\int_{0}^{\infty }\prod_{r_{k}\in R}\mathrm{Pr}\left[\min\left(\gamma_{1}g_{sr_{k}},\frac{\gamma_{2}g_{r_{k}d}}{\alpha^{2}\gamma_{2}g_{r_{k}b}x+1},\alpha^{2}\gamma_{2}g_{r_{k}b}x\frac{\gamma_{{th}_{1}}}{\gamma_{{th}_{2}}}\right)<\gamma_{th_{1}}\right]f_{g_{bd}}\left(x\right)dx$$

To remove the minimal operator in (A9), (A9) can be reformulated as

(A10) $$P_{\mathrm{out},bl}^{\mathrm{proact}}=\int_{0}^{\infty }\prod_{r_{k}\in R}\left\{1-\mathrm{Pr}\left[\gamma_{1}g_{sr_{k}}\geq \gamma_{th_{1}}\right]\cdot \mathrm{Pr}\left[\frac{\gamma_{2}g_{r_{k}d}}{\alpha^{2}\gamma_{2}g_{r_{k}b}x+1}\geq \gamma_{th_{1}},\alpha^{2}\gamma_{2}g_{r_{k}b}x\geq \gamma_{th_{2}}\right]\right\}f_{g_{bd}}\left(x\right)dx$$

Considering the correlation between the first term and the second term in the second probability operator, we have

(A11) $$P_{\mathrm{out},bl}^{\mathrm{proact}}=\int_{0}^{\infty }\prod_{r_{k}\in R}\left\{1-\mathrm{Pr}\left[\gamma_{1}g_{sr_{k}}\geq \gamma_{th_{1}}\right]\cdot \int_{\frac{\gamma_{th_{2}}}{\alpha^{2}\gamma_{2}x}}^{\infty }\mathrm{Pr}\left[\frac{\gamma_{2}g_{r_{k}d}}{\alpha^{2}\gamma_{2}yx+1}\geq \gamma_{th_{1}}\right]f_{g_{r_{k}b}}\left(y\right)dy\right\}f_{g_{bd}}\left(x\right)dx$$

Let $X=g_{bd}$, $Y=g_{r_{k}b}$, $Z_{k}=\min\left\{\gamma_{1}g_{sr_{k}},and\;\frac{\gamma_{2}g_{r_{k}d}}{\alpha^{2}\gamma_{2}yx+1}\right\}$ with given *x* and *y*. Then, after some straightforward steps, the $P_{\mathrm{out},bl}^{\mathrm{proact}}$ can be expressed by Equation (27).
